# Tobacco Harm Reduction as a Path to Restore Trust in Tobacco Control

**DOI:** 10.3390/ijerph18115560

**Published:** 2021-05-22

**Authors:** Tamar M. J. Antin, Geoffrey Hunt, Rachelle Annechino

**Affiliations:** Center for Critical Public Health, the Institute for Scientific Analysis, Alameda, CA 94501, USA; huntgisa@ix.netcom.com (G.H.); rachelle@criticalpublichealth.org (R.A.)

**Keywords:** nicotine and tobacco, tobacco control, tobacco prevention, institutional trust, harm reduction

## Abstract

The controversy of tobacco harm reduction in the United States persists despite evidence that an important audience of tobacco prevention and control, i.e., the people who use or are likely to use nicotine and tobacco products, are engaging in practices that may be considered harm reduction. Despite this, a significant proportion of the US tobacco control and prevention field continues to be guided by a precept that there is “no safe tobacco,” therefore failing to acknowledge practices that may be used to reduce the harms associated with consuming combustible forms of nicotine and tobacco. In this commentary, we argue that ignoring the potential benefits of harm reduction strategies may unintentionally lead to an erosion of trust in tobacco control among some members of the public. Trust in tobacco control as an institution is crucial for the success of tobacco control efforts. To ensure trust, we must return to our basic principles of doing no harm, developing programs that are responsive to people’s experiences, and providing resources in assisting people to reduce the harms that may be associated with practices, such as smoking, which adversely affect health. Only by respecting an individual’s priorities can we cultivate trust and develop tobacco prevention efforts that are grounded in the realities of people’s lives and responsive to their needs.

Accumulating evidence suggests that non-combustible tobacco products such as e-cigarettes, though not entirely risk-free, are relatively less harmful compared to those that are combustible and, therefore, reducing or replacing cigarette smoking with a non-combustible product such as e-cigarettes arguably yields a net benefit to one’s health [1,2,3,4,5,6]. This approach to tobacco prevention and control, involving substituting more harmful forms of nicotine for those that are less harmful, is referred to as tobacco harm reduction [7,8,9,10]. Though some current approaches to tobacco prevention, treatment, and control may be considered harm reduction (e.g., nicotine replacement therapies, smoke-free ordinances) [11], most often, tobacco prevention efforts in the United States are shaped by the precept that “there is no safe tobacco” [12] and any discussion of reducing harm remains controversial, particularly when it comes to young people [7,8,13,14]. Though it is true that no form of nicotine is free of risk, this precept fails to accommodate the circumstances of those adults and young adults who would otherwise smoke cigarettes and who may be (or could be) motivated to reduce the harms associated with combustible tobacco products but are not yet willing or interested in quitting nicotine use entirely [15,16]. While the public health community remains embroiled in debates about tobacco harm reduction [1,8,9,10], emerging evidence suggests that harm reduction strategies are nevertheless being practiced by many people who use nicotine and tobacco products [13,14,15,16]. Sadly, this adoption is occurring with little guidance (and conflicting messaging) from U.S. public health agencies responsible for tobacco prevention.

Among the troubling consequences associated with a failure to acknowledge the potential of tobacco harm reduction is the distrust that it may engender among the publics who use nicotine and tobacco products. Tobacco prevention messages embedded solely within a “no safe tobacco” framework not only confuse the current scientific evidence about the relative risk of nicotine and tobacco products [1,2,3,4,5,6,9,12], but they also delegitimize the practice of tobacco harm reduction and the actual strategies that people use to navigate the risks related to smoking cigarettes and other combustible products. These strategies include breaking cigarettes in half prior to smoking or adding nicotine vaping to reduce smoking frequency—both especially salient examples from our multiple studies of nicotine and tobacco use among diverse populations of adults and young adults in California (T2; [15,16]) More recently, some of our participants have described replacing nicotine vaping with smoking cigarettes because they believed that cigarettes were less dangerous than vaping—a belief that does not reflect current scientific consensus, yet one that can be easily understood in light of some public health prevention campaigns in the state of California. Disregarding tobacco harm reduction as one justifiable strategy for minimizing tobacco-related risks may fail to honor and support the lived experiences of many people, who use nicotine and tobacco, while also creating conflict between tobacco prevention messages and the available scientific evidence on relative risk—two pitfalls that could jeopardize trust in tobacco control institutions. 

The COVID-19 pandemic has revealed just how important the public’s trust in public health institutions is. Without trust, compliance with public health recommendations and mandates may be low [17,18]. Institutional trust has long been acknowledged as crucial for ensuring the success of public health programs including tobacco control programs where the level of trust in tobacco control among the public has important implications for understanding how the public receives and responds to anti-tobacco messaging [19,20,21,22]. Though definitions of trust are hotly debated [23,24,25], trust generally involves a relationship between a truster (e.g., the public) and a trustee (e.g., tobacco control), and refers to the truster holding “confident positive expectations” about the trustee ([26], p. 439). A review of the literature on trust in public institutions suggests five characteristics that are important for establishing institutional trust: (1) credible commitment (i.e., consistently acting in the public’s interest), (2) benevolence (i.e., demonstrating “care and concern” for the public through its actions (p. 625), (3) honesty (i.e., demonstrating altruism and “adherence to ethical standards” (p. 626), (4) competency (i.e., effective performance and productivity), and (5) fairness (i.e., adhering to equitable principles) [24].

Unfortunately, trust in public health appears to have eroded in recent decades [27] which scholars suggest may be due to: (1) a lack of trust in general of public institutions in the US, which has intensified in recent years; (2) the media’s participation in exaggerating public health risk; (3) high profile examples of public health issues where public health officials have been wrong, misleading, and/or perceived as protecting the wrong interests (e.g., politics over the interests of the public, ambiguous pandemic response); (4) perceptions that regulatory agencies have failed to act in the interests of the public, and (5) a decline in respect in scientific and medical expertise among the public [27,28,29]. The erosion of trust in public health institutions is an increasingly important issue today, in light of public health’s precarious position in the public’s consciousness due to the COVID-19 pandemic [17,30,31,32]. Given that trust in one sector of a system may have cascading effects for other sectors within the system [33], declines in trust related to the pandemic may have troubling consequences for many public health agencies, including tobacco control agencies.

To date, very little research has examined trust in government agencies responsible for tobacco prevention and cessation. In a national survey designed to examine perceptions of the FDA, the federal agency charged with regulating tobacco products, Boynton and colleagues [34] found that a majority of people who smoke (66.6%) “believed that the FDA can effectively regulate tobacco products,” but that their general trust in the federal government was relatively low. Case and colleagues [35], using data from the 2015 Health Information National Trends Survey-FDA Survey, found that people who reported ever using e-cigarettes reported significantly less trust in government health organizations compared to people who reported never using e-cigarettes. A small focus group study in North Carolina, examining perceptions of trust in federal agencies responsible for tobacco prevention messaging, highlighted ambivalent narratives related to trust among adults [19]. In particular, their analysis revealed that perceived integrity and competence of the agency, beliefs about the agency’s motives, how the media portrays the agency, and the extent of familiarity with the agency were all factors contributing to trust/distrust [36]. These themes are consistent within the broader literature on the factors important for establishing institutional trust [24,37]. In a formative qualitative study designed to inform program planning for the Connecticut Tobacco Control Program, McCullough and colleagues [38] conducted focus groups with primarily low SES adults who reported currently smoking. Though not explicitly focused on trust in tobacco control itself, their analysis suggested that participants distrusted tobacco control and held “negative attitudes about the tobacco industry’s and government entities’ perceived roles in sponsoring tobacco control campaigns” collaboratively, rendering ads untrustworthy and calling into question the motivations of tobacco control ([38] p. 553). As trust in tobacco control is dependent in part upon perceptions of benevolence—the notion that the government is acting in the best interest of the public ([24], p. 625)—these preliminary findings, if widespread, have serious implications for the effectiveness of tobacco control efforts.

Despite the best intentions of tobacco control agencies, emerging evidence suggests that trust in tobacco control among those who are among tobacco control’s most important audiences—i.e., people who use nicotine and tobacco products or who are likely to start—may be low. Preliminary analysis from our ongoing study investigating perceptions of tobacco harm reduction among LGBTQ+ young adults in California (TRDRP Grant # T30IR0890) highlights young adults’ skepticism about available health information on the relative risk of cigarettes and nicotine vaping devices, a theme which may have important implications for young people’s trust in tobacco control. Narratives from some participants suggest that public health information that evades questions of the relative risk of vaping nicotine compared to smoking cigarettes combined with a lack of interest in vaping, led some participants to continue smoking despite a desire to reduce their risk from tobacco products. The extent to which LGBTQ+ young adults perceive that this ambiguity in health information is related to low trust or distrust in tobacco control is not yet known.

More than ever, reflecting on ways to improve our own practice in public health is crucial, and part of that may be “returning to our discipline’s basic principles” of doing no harm [30]. Anti-tobacco messages, based on the precept that there is “no safe tobacco”, are at risk of not merely stigmatizing nicotine and tobacco use but more importantly those who use [39,40,41]. Instead, we must reimagine tobacco prevention efforts to respond in a nonjudgmental and compassionate way to those who are the intended recipients of tobacco prevention efforts. This could mean many different things, but one significant and important change would be to acknowledge that harm reduction strategies are valid and important for protecting the health of all. As Reuter and Caulkins [42] argued in 1995 in support of drug policy harm reduction: “the rhetorical and policy-oriented emphasis on making drug use less acceptable and drugs less available, as well as the focus on drug prevalence as the dominant indicator of program success, has probably outlived its usefulness” ([42], pp. 1059–1060; [43], p. 780). Twenty-five years later, this argument is still relevant and should be applied to the contemporary tobacco control arena, where efforts to increase the social unacceptability of smoking dominate the agenda and where harm reduction strategies remain, in some circles, simply unacceptable. Yet, harm reduction strategies are critically important because they are based on the reality that people, when adopting certain practices, weigh competing priorities in their decision-making processes, and health is just one of many. Harm reduction is an approach that is responsive to people’s experiences and can provide resources in assisting people to reduce the harms that may be associated with practices, such as smoking, which adversely affect health [43]. Only by respecting an individual’s health priorities can we cultivate trust and develop tobacco control efforts that are grounded in the realities of people’s lives and responsive to their needs. As agents tasked with creating a positive, supportive, and responsive public health system, that is our job.

## References

[1] Abrams D.B., Glasser A.M., Pearson J.L., Villanti A.C., Collins L.K., Niaura R.S. (2018). Harm minimization and tobacco control: Reframing societal views of nicotine use to rapidly save lives. Annu. Rev. Public Health.

[2] Chan G.C.K., Stjepanović D., Lim C., Sun T., Shanmuga Anandan A., Connor J.P., Gartner C., Hall W.D., Leung J. (2021). A systematic review of randomized controlled trials and network meta-analysis of e-cigarettes for smoking cessation. Addict. Behav..

[3] Eaton D., Kwan L., Stratton K. (2018). Public Health Consequences of E-Cigarettes: Health and Medicine Division.

[4] Glynn T.J., Hays J.T., Kemper K. (2021). E-cigarettes, harm reduction, and tobacco control: A path forward?. Mayo Clin. Proc..

[5] Hartmann-Boyce J., McRobbie H., Lindson N., Bullen C., Begh R., Theodoulou A., Notley C., Rigotti N.A., Turner T., Butler A.R. (2020). Electronic cigarettes for smoking cessation. Cochrane Database Syst. Rev..

[6] Malas M., van der Tempel J., Schwartz R., Minichiello A., Lightfoot C., Noormohamed A., Andrews J., Zawertailo L., Ferrence R. (2016). Electronic cigarettes for smoking cessation: A systematic review. Nicotine Tob. Res..

[7] Eversman M.H. (2015). Tobacco Harm Reduction: An Emerging Health Issue for Social Work. J. Soc. Work Pract. Addict..

[8] Kozlowski L.T. (2017). Minors, moral psychology, and the harm reduction debate: The case of tobacco and nicotine. J. Health Polit. Policy Law.

[9] Kozlowski L.T., Abrams D.B. (2016). Obsolete tobacco control themes can be hazardous to public health: The need for updating views on absolute product risks and harm reduction. BMC Public Health.

[10] Warner K.E. (2018). How to Think—Not Feel—About Tobacco Harm Reduction. Nicotine Tob. Res..

[11] O’Connor R.J., Cummings K.M., Quah S.R. (2017). Tobacco harm minimization. International Encyclopedia of Public Health.

[12] Kozlowski L.T., Sweanor D.T. (2018). Young or adult users of multiple tobacco/nicotine products urgently need to be informed of meaningful differences in product risks. Addict. Behav..

[13] Eversman M.H. (2015). Harm reduction in U.S. tobacco control: Constructions in textual news media. Int. J. Drug Policy.

[14] Stimson G.V. (2016). A tale of two epidemics: Drugs harm reduction and tobacco harm reduction in the United Kingdom. Drugs Alcohol Today.

[15] Antin T., Hunt G., Kaner E., Lipperman-Kreda S. (2019). Youth perspectives on concurrent smoking and vaping: Implications for Tobacco 21 laws. Int. J. Drug Policy.

[16] Antin T.M.J., Hess C., Kaner E., Lipperman-Kreda S., Annechino R., Hunt G. (2020). Pathways of nicotine product use: A qualitative study of youth and young adults in California. Nicotine Tob. Res..

[17] Bargain O., Aminjonov U. (2020). Trust and Compliance to Public Health Policies in Times of COVID-19. J. Public Econ..

[18] Lovari A. (2020). Spreading (Dis)trust: COVID-19 misinformation and government intervention in Italy. Media Commun..

[19] Jarman K.L., Ranney L.M., Baker H.M., Vallejos Q.M., Goldstein A.O. (2017). Perceptions of the food and drug administration as a tobacco regulator. Tob. Regul. Sci..

[20] Schmidt A.M., Ranney L.M., Pepper J.K., Goldstein A.O. (2016). Source credibility in tobacco control messaging. Tob. Regul. Sci..

[21] Avery E.J. (2010). The role of source and the factors audiences rely on in evaluating credibility of health information. Public Relat. Rev..

[22] Pornpitakpan C. (2004). The persuasiveness of soure credibility: A critical review of five decades’ evidence. J. Appl. Soc. Psychol..

[23] Ozawa S., Sripad P. (2013). How do you measure trust in the health system? A systematic review of the literature. Soc. Sci. Med..

[24] Kim S.-E. (2016). The role of trust in the modern administrative state: An integrative model. Adm. Soc..

[25] Shockley E., Neal T.M.S., PytlikZillig L.M., Bornstein B.H. (2016). Interdisciplinary Perspectives on Trust: Towards Theoretical and Methodological Integration.

[26] Lewicki R.J., McAllister D.J., Bies R.J. (1998). Trust and distrust: New relationships and realities. Acad. Manag. Rev..

[27] Cummings L. (2014). The “trust” heuristic: Arguments from authority in public health. Health Commun..

[28] Camargo K., Grant R. (2015). Public health, science, and policy debate: Being right is not enough. Am. J. Public Health.

[29] Rădoi M., Lupu A., Maturo A., Hošková-Mayerová Š., Soitu D.-T., Kacprzyk J. (2017). Understanding institutional trust. What does it mean to trust the health system?. Recent Trends in Social Systems: Quantitative Theories and Quantitative Models.

[30] Barocas J., Gandhi M. Harm Reduction Principles Can Help Us Restore Trust in Public Health Messaging on COVID-19. https://blogs.bmj.com/bmj/2020/12/15/harm-reduction-principles-can-help-us-restore-trust-in-public-health-messaging-on-covid-19/.

[31] Erdman S.L. A “Building Distrust” in Public Health Agencies Is “The Elephant in the Room”, Fauci Says—CNN. https://www.cnn.com/2020/10/22/health/fauci-distrust-building-coronavirus/index.html.

[32] Ward P.R. (2020). A sociology of the COVID-19 pandemic: A commentary and research agenda for sociologists. J. Sociol..

[33] Searle R., Nienaber A.-M., Sitkin S.B., Searle R.H., Nienaber A.-M.I., Sitkin S.B. (2018). Introduction. The Routledge Companion to Trust.

[34] Boynton M.H., Agans R.P., Bowling J.M., Brewer N.T., Sutfin E.L., Goldstein A.O., Noar S.M., Ribisl K.M. (2016). Understanding how perceptions of tobacco constituents and the FDA relate to effective and credible tobacco risk messaging: A national phone survey of U.S. adults, 2014–2015. BMC Public Health.

[35] Case K.R., Lazard A.J., Mackert M.S., Perry C.L. (2018). Source credibility and E-cigarette attitudes: Implications for tobacco communication. Health Commun..

[36] Ranney L.M., Jarman K.L., Baker H.M., Vu M., Noar S.M., Goldstein A.O. (2018). Factors influencing trust in agencies that disseminate tobacco prevention information. J. Prim. Prev..

[37] Mayer R.C., Davis J.H., Schoorman F.D. (1995). An integrative model of organizational trust. Acad. Manag. Rev..

[38] McCullough A., Meernik C., Baker H., Jarman K., Walsh B., Goldstein A.O. (2018). Perceptions of tobacco control media campaigns among smokers with lower socioeconomic status. Health Promot. Pract..

[39] Bell K., McCullough L., Salmon A., Bell J. (2010). Every space is claimed: Smokers’ experiences of tobacco denormalisation. Sociol. Health Illn..

[40] Graham H. (2012). Smoking, stigma and social class. J. Soc. Policy.

[41] Voigt K. (2010). Smoking and social justice. Public Health Ethics.

[42] Reuter P., Caulkins J.P. (1995). Redefining the goals of national drug policy: Recommendations from a working group. Am. J. Public Health.

[43] Marlatt G.A. (1996). Harm reduction: Come as you are. Addict. Behav..

